# Episiotomy and its relationship to various clinical variables that influence its performance

**DOI:** 10.1590/1518-8345.0334.2686

**Published:** 2016-05-20

**Authors:** Carmen Ballesteros-Meseguer, César Carrillo-García, Mariano Meseguer-de-Pedro, Manuel Canteras-Jordana, Mª Emilia Martínez-Roche

**Affiliations:** 1 PhD, Associate Professor, Facultad de Enfermería, Universidad de Murcia, Murcia, Spain. Nurse-midwife, Hospital Clínico Universitario Virgen de la Arrixaca del Servicio Murciano de Salud, Murcia, Spain; 2 PhD, Associate Professor, Facultad de Enfermería, Universidad de Murcia, Murcia, Spain. Chief section, Dirección General de Recursos Humanos del Servicio Murciano de Salud, Murcia, Spain; 3 PhD, Professor, Departamento de Psiquiatría y Psicología Social, Facultad de Psicología, Universidad de Murcia, Murcia, Spain; 4 MD, PhD, Full Professor, Departamento de Bioestadística, Facultad de Enfermería, Universidad de Murcia, Murcia, Spain; 5 PhD, Full Profesor, Departamento de Enfermería, Facultad de Enfermería, Universidad de Murcia, Murcia, Espanha. Head of studies Unidad Docente Obstétrico-Ginecológica (Matrona), Murcia, Espanha

**Keywords:** Episiotomy, Natural Childbirth, Obstetric Nursing, Obstetric Surgical Procedures, Labor Obstetric

## Abstract

**Objective:**

to understand the episiotomy rate and its relationship with various clinical variables.

**Method:**

a descriptive, cross-sectional, analytic study of 12,093 births in a tertiary hospital. Variables: Parity, gestational age, start of labor, use of epidural analgesia, oxytocin usage, position during fetal explusion, weight of neonate, and completion of birth. The analysis was performed with SPSS 19.0.

**Results:**

the global percentage of episiotomies was 50%. The clinical variables that presented a significant association were primiparity (RR=2.98), gestational age >41 weeks (RR=1.2), augmented or induced labor (RR=1.33), epidural analgesia use (RR=1,95), oxytocin use (RR=1.58), lithotomy position during fetal expulsion (RR=6.4), and instrumentation (RR=1.84). Furthermore, maternal age ≥35 years (RR=0.85) and neonatal weight <2500 g (RR=0.8) were associated with a lower incidence of episiotomy.

**Conclusions:**

episiotomy is dependent on obstetric interventions performed during labor. If we wish to reduce the episiotomy rate, it will be necessary to bear in mind these risk factors when establishing policies for reducing this procedure.

## Introduction

Episiotomy is a surgical procedure to widen the inferior part of the vagina, the vulvar ring, and the perineal tissue during the fetal expulsion stage of birth^(^ 1 ^)^.

In Spain, according to data from 2005 onward, there is a wide variability in the percentage of episiotomies, from 33% to 73%, according to Autonomous Communities [Comunidades Autónomas]^(^ 2 ^)^. In the University Clinical Hospital of the Virgin of Arrixaca [Hospital Clínico Universitario Virgen de la Arrixaca], the rate is approximately 50% of all births. This statistic is far from the recommendations of the World Health Organization, which has set a maximum of 15%. The reasons for the variability in the rate of this procedure have not been established; consequently, the Health Ministry has developed the Strategy for Normal Birthing in the National Health System, which aims to bring birthing care in line with scientific evidence, among other goals. Various systematic reviews have concluded that the systematic use of episiotomy does not provide more benefits than restricted use^(^ 4 ^)^. 

The variations in episiotomy rates may be related to the variations in clinical practice related to common obstetric situations such first childbirth, instrument-assisted birth, and epidural use^(^ 5 ^-^ 6 ^)^.

An analysis of the obstetric variables in the database of the University Clinical Hospital of the Virgin of Arrixaca would be a useful tool for determining factors related to this variability in episiotomy rates.

The objective of the present study is to determine the episiotomy rate and its relationship with particular clinical variables.

## Method

**Figure 1:**
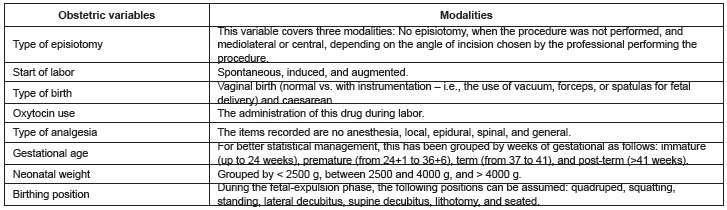
- Obstetrical variables collected for the study from clinical data. Murcia, Spain, 2012-2013

A descriptive, cross-sectional, analytic study was conducted using retrospective data collection. The initial sample was composed of 15,074 women with clinical records of births that occurred between January 1, 2011, and December 31, 2012, who received care at University Clinical Hospital of the Virgin of Arrixaca in Murcia, Spain. The data were collected from the hospital's clinical database (SELENE). Inclusion criteria were births that occurred in the hospital setting for which the relevant study data were available. Incomplete episodes, births outside of hospitals, caesareans, and cases with errors introduced during registration were excluded. Finally, 12,093 birth episodes were analyzed. The study was reviewed by the Hospital Research Ethics Committee. The obstetric variables collected from the clinical data are shown in Figure 1.

The data were transformed and encoded using the statistical software SPSS and Excel. A descriptive analysis was performed on the obstetric variables (start of labor, episiotomy, completion of birth, oxytocin use, birthing position, neonatal weight, gestational age, and type of analgesia), followed by an analysis of the bivariate associations between episiotomy and the aforementioned clinical variables using the chi-square test (c^2)^ to study the relationships among qualitative variables. On a third level, a trivariate analysis was performed on the variables that were statistically significant (p<0.05) and those that may have been confounding factors, such as first childbirth, epidural use, and neonatal weight. To correct for the sample size effect (n=12,093), the estimated effect size was added using Cramer's V. The relative risk (RR) was also calculated for each pair of qualitative variables as a relative measure of the effect, to determine the magnitude or strength of the association between variables.

## Results

The episiotomy rate for all vaginal births in the study hospital was 50.4% in 2011 and 49.4% in 2012. Episiotomies were performed in 40.8% of all normal births in 2011 and in 36.5% in 2012. In instrumented births, the episiotomy rate was over 90% for all modalities (spatulas, vacuum, and forceps; Table 1).

**Table 1:** - Description of total births at University Clinical Hospital of the Virgin of Arrixaca in the years 2011-12 and the percentage of episiotomies. Murcia, Spain, 2012-2013

Procedures	2011	2012
Total births (vaginal + caesarean)	7,566	7,508
Vaginal births (normal + instrumented)	6,075	6,018
Episiotomies in vaginal births (%)	50.4	49.4
Normal births	4,649	4,531
Episiotomies in normal births (%)	40.8	36.5
Instrumented births	1,426	1,487
Episiotomies in instrumented births (%)	92.7	93

During birthing assistance, there is a specific set of variables that can influence the necessity of an episiotomy. The studied variables are parity, gestational age, start of labor (spontaneous, induced, or augmented), epidural analgesia use, oxytocin use, position during fetal expulsion, neonatal weight, and the completion of birth (normal or instrumented).

To study *parity*, the data were sorted by this item. The number of episiotomies was calculated for the primiparous women (2,450 [68.3%] in 2011 and 2,388 [70.3%] in 2012) and for the multiparous women (those with one or more previous births; 783 [27.1%] in 2011 and 653 [31%] in 2012). The tendency was for primiparous women to undergo an episiotomy and for multiparous women not to (RR=2.98).

The analysis of *gestational age* in relation to episiotomy showed a tendency to perform episiotomies for post-term births and a tendency not to perform one under other circumstances. The statistical test used showed significance (c^2^=52.810, p=0.0005), but a there was a low effect for the relationship (V=0.033). The RR of having an episiotomy in cases of post-term gestation (>41 weeks) was 1.2 compared with term births (37-41 weeks).

In analyzing *start of labor* in relation to episiotomy, it was observed that of the 7,061 pregnancies that began labor spontaneously, 45.5% included an episiotomy, compared with 61.5% of 2,852 pregnancies with induced labor, and 59.2% of 2,170 pregnancies with augmented labor. A comparison of these three groups revealed a statistically significant difference (c^2^=270.911, p>0.000), although Cramer's V showed that this relationship had a minor effect (V=0.140). The RR of episiotomy in births with interventions (induction or augmentation) was 1.33 in relation to spontaneous labor. 

Regarding the *type of analgesia*, the data showed a tendency toward episiotomy in births with epidural analgesia compared with those with no analgesia or with local (perineal) analgesia. The statistical test showed that the difference was significant (c^2^=1150.339; p<0.0005), with a moderate effect between the two variables (V=0.307). The RR of episiotomy in women who used epidural analgesia was 1.95.

The analysis of *oxytocin administration* during birth indicated a statistically significant relationship between episiotomy and oxytocin administration (c^2^=237.527, p<0.0005), although the association was low (V=0.138). The RR of episiotomy when oxytocin was administered during birth was 1.58 versus not using the drug.

The analysis of the *position during the fetal expulsion phase* showed a tendency to perform episiotomy when women were in the lithotomy position during this phase. For all other positions, the tendency was not to perform one. The statistical test used showed that the difference was significant (c^2^=236.515, p<0.0005). The RR of episiotomy in the lithotomy position was 6.4 with respect to other positions.

The analysis of *neonatal* *weight* in relation to episiotomy showed a tendency not to perform episiotomy with low-weight neonates <2,500 g while with those weighing 2,500-4,000 g and >4,000 g, the tendency is to perform one. The statistical test used showed significance (c^2^=84.157, p<0.0005) but with a low effect for the relationship (V=0.024). The RR of episiotomy with neonates <2,500 g is 0.8 compared with greater weights.

The statistical analysis of the data related to *instrumented birth* showed a tendency to perform an episiotomy in instrumented births (92.92%), while in normal births, the tendency was to not perform one. The statistical test showed significant differences (c^2^=2644.06; p=0.0005), and the relationship between the variables can be considered medium-high (V=0.464). The RR of episiotomy in instrumented births was 1.84 compared with normal births.

In an effort to decrease possible confounding factors, a trivariate analysis of episiotomy was performed using the factors that had previously shown statistically significance and had a medium-high effect size (i.e., instrumental birth and epidural analgesia use) and those variables that might constitute a confounding factor, such as primiparity, epidural use, and neonatal weight.

Similarly, the analysis of the variable *parity related to completion of birth and to use of episiotomy* showed a statistically significant relationship (c^2^= 1043.44; p<0.0005) in primiparous and in multiparous women (c^2^= 665.06; p<0.0005); there was a tendency to perform mediolateral episiotomies in instrumented births independent of parity and to not perform episiotomy in normal births.

Similarly, the statistical analysis of the variable *type of birth completion related to epidural use and episiotomy* showed a statistically significant relationship. Among women who did not use epidural anesthesia, there was a tendency to not perform an episiotomy in normal births and to perform one in instrumented births. The statistical test showed significant differences (c^2^=263.664; p=0.000) with a moderate relational effect (V=0.306). The results were similar for the women who used epidurals: the statistical test used showed a significant difference (c^2^=159.256; p=0.000) and a moderate relational effect (V=0.461).

Likewise, the statistical analysis of the variables *type of birth completion, neonatal weight, and episiotomy* was statistically significant for the births in which an episiotomy was performed (c^2^=22.224; p=0.000), with a low relational effect V=0.065. There was a tendency to perform an episiotomy in normal births of neonates with a weight below 2,500 g and in instrumented births of neonates with a weight greater than 4,000 g.

 For the variable *use of epidural analgesia related to parity and to episiotomy,* the statistical analysis showed a statistically significant relationship in primiparous (c^2^= 51.37, p<0.0005) and in multiparous (c^2^= 46.86, p<.0005) women in 2011 and in 2012 (primiparous c^2^=5.57, p<0.0005 and multiparous c^2^=62.07, p<0.0005). The tendency in the primiparous women was to perform an episiotomy in women who used an epidural and to not perform one in women who did not use an epidural. For the multiparous women, this tendency was also observed.

In this same manner, the statistical relationship between the variables *epidural use, neonatal weight, and episiotomy use* was statistically significant for the use of epidural analgesia (c^2^=8.321; p=0.016), although the relational effect was low, V=0.032. The observed tendency was to perform an episiotomy in mothers who used epidural anesthesia and whose neonates weighed more than 4,000 g. 

## Discussion

Our results show that *primiparity* is one of the main risk factors associated with episiotomy. This finding coincides with the findings of other authors^(^ 5 ^-^ 7 ^)^. This variable was considered a confounding factor because in clinical trials with parturient women, it should be controlled. Furthermore, episiotomy in primiparous women presents another implication, as it significantly and independently increases the risk of episiotomy and tears in subsequent births^(^ 8 ^)^.

*Gestational age* over 41 weeks constitutes another risk factor associated with episiotomy (RR=1.2). Post-term gestations comprised 12% of the studied births, and of these, 60% had an episiotomy, versus 44.5% of preterm births and 49.6% of term births. This statistic is explained by the tendency to induce labor in post-term births (c^2^=18.085, p>0.005) and by the tendency to complete these births with instrumentation (c^2^=36.315; p=0.02). This data is interesting as it confirms the existence of an "intervention cascade" when there are interventions in normal labor development. This finding was also confirmed by a Cochrane review regarding perineal care that concluded that the use of epidural analgesia increases the likelihood of instrumented birth and episiotomy, thus increasing the risk of perineal trauma ^(9)^.

In the analysis of *start of labor* in relation to episiotomy, we found that births that began spontaneously had a lower risk of episiotomy than those that were augmented or induced. Our results concur with the findings of other authors, although they associated this relationship with primiparous status^(^ 6 ^)^. Regarding the method labor induction or augmentation, our results showed a tendency to perform episiotomy in births in which oxytocin is administered versus births in which labor is allowed to evolve normally (54.4% vs 34.4%).

 In relation to the *type of completion of vaginal birth*, instrumentation can be considered a risk factor for episiotomy compared with a normal birth (93% vs 38.7%; RR=1.84). This statistic coincides with the findings of numerous authors^(^ 6 ^,^ 10 ^-^ 11 ^)^. The number of episiotomies and instrumented births increases with the complexity of the hospital. As in other specialties, this relationship arises from the referral of complex procedures to centers with greater technological and human resources^(^ 12 ^)^. The studied hospital delivered 23.4% of births with instrumentation in 2011 and 24.7% in 2012. The role of routine episiotomy in instrumented birth is not well studied and requires more research. It seems that its use is justified by the decrease of perineal tears, especially when forceps are used^(^ 13 ^)^.

Another variable that influences the use of episiotomy is the *type of analgesia* used during labor, specifically the use of epidural analgesia. In births in which the woman chooses an epidural as her method of pain relief, episiotomies are more often performed than in those in which no analgesia is used or in which the analgesia is local (58.4% versus 30%). These results coincide with the findings of other authors^(^ 6 ^-^ 7 ^,^ 11 ^,^ 14 ^-^ 16 ^)^. Moreover, this tendency is observed regardless of parity and the method of birth completion. Regarding neonatal weight >4000 g, we observed an association between epidural use and episiotomy that increased the risk of episiotomy in such cases compared with births without epidurals with the same neonatal weight (65.8% versus 26.3%)^(^ 17 ^-^ 18 ^)^. 

*Maternal position* during the fetal expulsion phase also affects whether an episiotomy will be performed; the lithotomy position was clearly associated with episiotomy compared with other positions (52.2% versus 30%). These results coincide with those of other authors, who also conclude that alternative positions (supine, seated, lateral, standing, squatting, and quadruped) are associated with less frequent use of episiotomy and that it should be left to women to choose the most comfortable position for giving birth^(^ 6 ^,^ 11 ^)^.

The results showed also protective factors in relation to episiotomy use, such as maternal age and fetal weight.

*Maternal age* greater than 35 years was associated with a decreased incidence of episiotomy (45.5%) compared with younger ages (54.4%). The reviewed studies suggest that use of episiotomy is not associated with maternal age^(^ 10 ^,^ 19 ^-^ 20 ^)^. This difference may be related to the increased maternal age in our study; the reviewed studies included women up to age 35 years, while our sample comprised a population aged 14 to 53 years, of which 25% were older than 35 years.

In relation to *neonatal weight*, the results of this study showed a decrease in the episiotomy rate when the neonate weighed <2,500 g (43%) compared with normal weights (53.4%) and weights greater than 4,000 g (57.2%). Other authors did not find this association between neonatal weight and episiotomy^(^ 11 ^)^. On the contrary, for heavier fetuses (weight >4,000 g), the results showed an increase in episiotomy risk associated with instrumented birth or the use of epidural analgesia ^(21)^.

Our research had some limitations that should be qualified. First, there was possible under-registration of clinical data. Furthermore, a larger sample size from hospitals of various levels would allow a greater generalization of the obtained results.

## Conclusion

The episiotomy rate at the studied hospital was higher than the recommendations of WHO, which has found that episiotomy is not an isolated procedure and is not independent of other obstetric practices. Instead, it is associated with particular clinical variables that can increase the rate of this procedure. These variables are primiparity (RR=2.97), lithotomy position during the fetal explusion phase (RR=6.4), epidural analgesia use (RR=1.95), instrumented birth (RR= 1.84), oxytocin use during labor (RR=1.58), labor induction (RR=1.33), and post-term births >41 weeks of gestation (RR=1.2).

In addition, there are factors that protect against the performance of episiotomy: fetal weight <2500 g (RR=0.8) and maternal age >35 years (RR=0.8). Fetal weight >4000 g alone is not a risk factor for episiotomy, but when it was associated with epidural use or instrumented birth, the risk of episiotomy was increased.

Given our results and as a practical implication of the study, if we wish to decrease the episiotomy rate, it will be necessary to bear in mind the factors that influence its practice, establish policies to reduce these procedures, and ensure that they are upon by all health professionals who assist women during the birthing process. 
